# Synthetic miR-145 mimic inhibits multiple myeloma cell growth *in vitro* and *in vivo*

**DOI:** 10.3892/or.2021.8125

**Published:** 2021-06-28

**Authors:** Qi Zhang, Weiqun Yan, Yang Bai, Hao Xu, Changhao Fu, Wenwen Zheng, Yingqiao Zhu, Jie Ma

Oncol Rep 33: 448-456, 2015; DOI: 10.3892/or.2014.3591

Following the publication of this paper, it was drawn to the Editors attention by a concerned reader that certain of the flow cytometric and western blotting data shown in Fig. 3A and C respectively, and the tumor images shown in Fig. 7A, bore unexpected similarities to data appearing in different form in other articles by different authors. Owing to the fact that some of the contentious data in the above article had already been published elsewhere, or were already under consideration for publication, prior to its submission to *Oncology Reports*, the Editor has decided that this paper should be retracted from the Journal. After having been in contact with the authors, they agreed with the decision to retract the paper. The Editor apologizes to the readership for any inconvenience caused.

